# Metabarcoding of mycetangia from the *Dendroctonus frontalis* species complex (Curculionidae: Scolytinae) reveals diverse and functionally redundant fungal assemblages

**DOI:** 10.3389/fmicb.2022.969230

**Published:** 2022-09-16

**Authors:** Karina Vazquez-Ortiz, Rosa María Pineda-Mendoza, Román González-Escobedo, Thomas S. Davis, Kevin F. Salazar, Flor N. Rivera-Orduña, Gerardo Zúñiga

**Affiliations:** ^1^Laboratorio de Variación Biológica y Evolución, Departamento de Zoología, Escuela Nacional de Ciencias Biológicas, Instituto Politécnico Nacional, Mexico City, Mexico; ^2^Laboratorio de Microbiología, Facultad de Zootecnia y Ecología, Universidad Autónoma de Chihuahua, Chihuahua, Mexico; ^3^Department of Forest and Rangeland Stewardship, Warner College of Natural Resources, Colorado State University, Fort Collins, CO, United States; ^4^Laboratorio de Ecología Microbiana, Departamento de Microbiología, Escuela Nacional de Ciencias Biológicas, Instituto Politécnico Nacional, Mexico City, Mexico

**Keywords:** mycetangia, mycobiome, ITS, symbiosis, bark beetles, *Dendroctonus*

## Abstract

*Dendroctonus*-bark beetles are associated with microbes that can detoxify terpenes, degrade complex molecules, supplement and recycle nutrients, fix nitrogen, produce semiochemicals, and regulate ecological interactions between microbes. Females of some *Dendroctonus* species harbor microbes in specialized organs called mycetangia; yet little is known about the microbial diversity contained in these structures. Here, we use metabarcoding to characterize mycetangial fungi from beetle species in the *Dendroctonus frontalis* complex, and analyze variation in biodiversity of microbial assemblages between beetle species. Overall fungal diversity was represented by 4 phyla, 13 classes, 25 orders, 39 families, and 48 genera, including 33 filamentous fungi, and 15 yeasts. The most abundant genera were *Entomocorticium*, *Candida*, *Ophiostoma-Sporothrix*, *Ogataea*, *Nakazawaea*, *Yamadazyma*, *Ceratocystiopsis*, *Grosmannia-Leptographium*, *Absidia*, and *Cyberlindnera*. Analysis of α-diversity indicated that fungal assemblages of *D*. *vitei* showed the highest richness and diversity, whereas those associated with *D*. *brevicomis* and *D*. *barberi* had the lowest richness and diversity, respectively. Analysis of β-diversity showed clear differentiation in the assemblages associated with *D*. *adjunctus*, *D*. *barberi*, and *D*. *brevicomis*, but not between closely related species, including *D*. *frontalis* and *D*. *mesoamericanus* and *D*. *mexicanus* and *D*. *vitei*. A core mycobiome was not statistically identified; however, the genus *Ceratocystiopsis* was shared among seven beetle species. Interpretation of a tanglegram suggests evolutionary congruence between fungal assemblages and species of the *D*. *frontalis* complex. The presence of different amplicon sequence variants (ASVs) of the same genus in assemblages from species of the *D*. *frontalis* complex outlines the complexity of molecular networks, with the most complex assemblages identified from *D*. *vitei*, *D*. *mesoamericanus*, *D*. *adjunctus*, and *D*. *frontalis*. Analysis of functional variation of fungal assemblages indicated multiple trophic groupings, symbiotroph/saprotroph guilds represented with the highest frequency (∼31% of identified genera). These findings improve our knowledge about the diversity of mycetangial communities in species of the *D*. *frontalis* complex and suggest that minimal apparently specific assemblages are maintained and regulated within mycetangia.

## Introduction

*Dendroctonus* bark beetles (Curculionidae: Scolytinae) are agents of disturbance in coniferous forest in North and Central America, and their populations occasionally outbreak to affect large areas of healthy forest with consequences for biodiversity, ecosystem and landscape degradation, and economic damage to timber resources (Six and Bracewell, 2015). Beetles breed in the inner bark and feed on the phloem of living trees of genera *Pinus*, *Larix*, *Pseudotsuga*, and *Piceae* (Family: Pinaceae). This strategy requires them to have metabolic and nutritional capabilities to overcome chemical defenses of trees and exploit a substrate rich in complex polysaccharides (e.g., cellulose, hemicellulose, starch, pectin) and other polymers (e.g., lignin) that are not readily available to them (Krokene, 2015; Chiu et al., 2019; Pace, 2019; Soto-Robles et al., 2021). *Dendroctonus* bark beetles are associated with facultative microbes (e.g., filamentous fungi, yeasts, bacteria) that may aid them in the tasks of detoxifying terpenes (DiGuistini et al., 2011; Adams et al., 2013; Xu et al., 2015; Dai et al., 2022), degrading complex molecules (Valiev et al., 2009; Cano-Ramírez et al., 2016; Briones-Roblero et al., 2017a), uric acid recycling and fixing nitrogen (Ayres et al., 2000; Bleiker and Six, 2007; Goodsman et al., 2012; Morales-Jiménez et al., 2012, 2013), producing semiochemicals (Boone et al., 2013; Cale et al., 2019), and regulating ecological interactions between symbionts and pathogens (Cardoza et al., 2006; Adams et al., 2009; Cheng et al., 2015, 2018; Xu et al., 2016).

Microbial communities are acquired by *Dendroctonus* beetles *via* feeding and contact during their development under the bark of trees and transported in the gut, on the integument, or in specialized secretory structures termed “mycetangia” (Vega and Biedermann, 2020). Mycetangia are compartmentalized cuticular spaces whose microenvironment changes throughout the beetle life cycle (Beaver, 1989; Six, 2012). These structures are common in ambrosia and bark beetles (Jordal and Cognato, 2012); however, in the genus *Dendroctonus*, they vary in their shape and location. For example, mycetangia occur as pits in *D*. *pseudotsugae* Hopkins, 1905 (Lewinsohn et al., 1994), pouches in the maxillary cardines in *D*. *ponderosae* Hopkins, 1902 and *D*. *jeffreyi* Hopkins, 1909 (Whitney and Farris, 1970), and as well-developed prothoracic sacs in the species of the *Dendroctonus frontalis* complex, including *D*. *adjunctus* Blandford, 1897, *D*. *approximatus* Dietz, 1890, *D*. *barberi* Hopkins, 1909, *D*. *brevicomis* LeConte, 1876, *D*. *frontalis* Zimmermann, 1868, *D*. *mexicanus* Hopkins, 1905, *D*. *mesoamericanus* Armendáriz-Toledano and Sullivan, 2015 and *D*. *vitei* Wood, 1974 (Barras and Perry, 1971; Yuceer et al., 2011; Six and Bracewell, 2015).

It is assumed that the mycetangia in bark beetles that feed on phloem evolved independently of nutritional requirements, unlike other insects that are nutritionally and physiologically dependent on fungi carried in these structures (Birkemoe et al., 2018; Vega and Biedermann, 2020). Mycetangia secrete specific amino acids, sterols and fatty acids that stimulate germination of fungal spores or hyphal growth (Barras and Perry, 1971; Happ et al., 1971; Yuceer et al., 2011; Six, 2012). These characteristics suggest that mycetangia should be highly selective and constrain the presence of microbes to specific taxa whose physiological capabilities are able to survive in this selective microenvironment (Beaver, 1989). However, it is also possible that phylogenetically unrelated microbes may have adapted to co-exist with symbiotic species within mycetangia (Six, 2012), and such adaptations could favor microbial dispersal within and between habitats.

It has been demonstrated that mycetangial microbes have a certain degree of specificity within *Dendroctonus* species (Harrington, 2005; Bracewell and Six, 2014; Birkemoe et al., 2018; Bracewell et al., 2018). Yet, few studies attempt to broadly evaluate composition of whole fungi and yeast assemblages, and culture-dependent approaches have limited taxonomic coverages. In this study, we comprehensively characterize the diversity of mycetangial assemblages from species in the *D. frontalis* complex using high-throughput next generation sequencing (NGS) of the internal transcribed spacer 2 region (ITS-2). Application of NGS has enhanced the characterization of microbial communities associated with *Dendroctonus* species and their host trees, and enables the evaluation of community change across developmental stages and growth conditions (e.g., field-grown whole adults, larvae, phloem, galleries, and laboratory-reared whole adults) (Durand et al., 2015, 2019; Dohet et al., 2016; Briones-Roblero et al., 2017b; Hernández-García et al., 2017; Cheng et al., 2018; Gonzalez-Escobedo et al., 2019). Here, we asked three fundamental questions about the diversity of microbes present in mycetangial structures: How diverse are the mycetangial assemblages of beetles in the *D*. *frontalis* complex? How specific are these assemblages to different beetle species? What are the potential functional roles of these assemblages? Accordingly, we test the following two respective hypotheses: (1) beetle species have similar mycetangial assemblages in terms of community composition, diversity, and trophic relationships, and (2) microbial assemblages show a high degree of specificity to beetle species given the dependence of these on filamentous fungi and the different environmental conditions and unique relationships with their host-trees. This study provides new insight into the biodiversity and function of bark beetle-fungal interactions and may have consequences for our understanding of complex multi-partner symbioses.

## Materials and methods

### Collection of insects

The species of the *Dendroctonus frontalis* complex (Lanier et al., 1988), including *Dendroctonus adjunctus*, *D*. *barberi*, *D*. *brevicomis*, *D*. *frontalis*, *D*. *mesoamericanus*, *D*. *mexicanus*, and *D*. *vitei* were collected in different localities from Mexico and United States (Table 1). Sterile forceps were used to directly remove pioneer adults from galleries built under the bark from five pine trees (∼15 m tall, ∼25 cm diameter) during the onset of colonization, but in the case from *D. barberi* and *D*. *brevicomis* beetles were collected from Lindgren funnel traps baited with endo-brevicomin (+) and α-pinene, and exo-brevicomin (+) and α-pinene (Synergy Canada Inc.), respectively. Insects collected in Mexico were stored and transported in sterile Magenta™ vessels GA-7 (Sigma-Aldrich, United States) at 4°C and processed immediately upon arrival at the laboratory, while those from the United States were shipped by air in vials of 70% alcohol. Species identification was carried out according to Armendáriz-Toledano and Zúñiga (2017).

**TABLE 1:** Collection sites from the species of the *Dendroctonus frontalis* complex.

Species	Location	Coordinates			Host/Collection method
		Latitude (*N*)	Longitude (W)	Altitude (m)	
***D*. *adjunctus***	Nevado de Colima, Jalisco^3^	19°35′06.0″	103°36′14.4″	4,260	*Pinus hartwegii*, at hand of attacked trees
***D*. *barberi***	Santa Fe National Forest, Nuevo México^2^	35°47′42″	106°36′21.6″	2,468	Unknown, Pheromone-baited Lindgren Funnel Trap
***D*. *brevicomis***	Cedar Pine Park, California^1^	34°15′36.5″	117°19′41.4″	1,552	Unknown, Pheromone-baited Lindgren Funnel Trap
	Farragut State Park, Idaho^2^	47°58′0.06″	116°34′25.4″	702	Unknown, Pheromone-baited Lindgren Funnel Trap
***D*. *frontalis***	El Madroño, Querétaro^3^	21°16′49.2″	99°08′53.6″	1,687	*P*. *teocote*, at hand of attacked trees
***D*. *mexicanus***	El Durazno, Guanajuato^3^	21°19′18.19″	99°47′5.494″	2,454	*P*. *teocote*, at hand of attacked trees
	Anteojitos, Nuevo León^1^	24°11′57.3″	99°54′11.5″	2,109	*P*. *cembroides*, at hand of attacked trees
***D*. *mesoamericanus***	Laguna de Montebello, Chiapas^3^	16°07′00″	91°42′00″	1,500	*P*. *oocarpa*, at hand of attacked trees
***D*. *vitei***	Cilantrillo, Nuevo León^3^	25°21′22.2″	100°19′32.7″	1,844	*P*. *cembroides*, at hand of attacked trees

The superscripts in the locality names represent the biological replicates of insects obtained at each site.

All insects were superficially disinfected by serial immersion in the following solutions at 1 min intervals: detergent solution (10 mmol L^–1^ Tris-HCl pH 8, 1 mmol L^–1^ EDTA, 10 mmol L^–1^ NaCl, 1% SDS, 2% Triton X-100), 70% ethanol solution and sterile distilled water. The head and abdomen of all insects were removed and placed in a sterile phosphate-buffer solution (PBS pH 7.4; 137 mmol L^–1^ NaCl, 2.7 mmol L^–1^ KCl, 10 mmol L^–1^ NaHPO_4_, 2 mmol L^–1^ KH_2_PO_4_). Under a stereoscopic microscopy, the portion of the prothorax (callus pronotal) containing the complete female mycetangium was cleaned directly again with detergent solution and 96% ethanol, using a camelhair brush. To maintain sterile conditions, dissections and surface sterilizations were performed in a laminar flow hood. Lastly, three replicates of 50 mycetangia of each sample (150/species) were transferred into 1.5 mL microtubes containing 200 μL of sterile PBS and stored at −20°C until DNA extraction. For *D. mexicanus* and *D. barberi* there were four (200 total mycetangia) and two (100 total mycetangia) replicates analyzed.

### DNA extraction, amplification, and sequencing

All samples were centrifuged for 5 min at 13,000 rpm to concentrate pronota, and supernatants were discarded. To each sample, 500 μL of lysis buffer (10 mM Tris-HCl pH 8, 1 mM EDTA, 10 mM NaCl, 1% SDS, 2% Triton X-100) were added, and later each pronotum set was macerated using sterile plastic pestles and vortexed with 0.2 g of zirconia beads for 1.5 min. Thereafter, 40 μL of Proteinase K (20 mg mL^–1^) were added and incubated at 60°C for 3 h. After incubation, 500 μL of chloroform-isoamyl alcohol (24:1 v/v) were added to the samples and mixed gently by inversion. The tubes were kept at −70°C for 1 h. Then 180 μL of 1% CTAB (hexadecyltrimethylammonium bromide) in 0.7 M NaCl and 80 μL of 5 M NaCl were added, and the samples were centrifuged for 5 min at 5,000 × *g* and the aqueous phase recovered in new sterile tubes. DNA was precipitated with 500 μL of 100% ethanol. DNA pellets were resuspended in sterile deionized water, and their concentration and purity were evaluated in a NanoDrop 2000c Spectrophotometer (Thermo Scientific, Wilmington, DE, United States). Universal primer pairs ITS3 (5′-GCA TCG ATG AAG AAC GCA GC-3′) and ITS4 (5′-TCC TCC GCT TAT TGA TAT GC-3′) were used to characterize the fungal assemblages of mycetangia samples. The ITS2 region was sequenced using paired-end 2 × 300 bp on an Illumina MiSeq sequencer at Macrogen Inc. (Seoul, Korea).

### Quality control and taxonomic assignment

Raw paired-end reads were imported into Quantitative Insights Into Microbial Ecology QIIME2 v.2022.2 (Bolyen et al., 2019). Sequences were quality filtered, trimmed, denoised, and merged using DADA2 plugin (Callahan et al., 2016). Chimeric sequences, singletons, and doubletons were identified and removed *via* the consensus method in DADA2. Representative ASVs were aligned with MAFFT and used for phylogenetic reconstruction in FastTree using plugins alignment and phylogeny. A pretrained Naïve Bayes classifier based on UNITE database (Kõljalg et al., 2020)^1^ at 97% similarity threshold was applied to assign the taxonomy. The taxonomic identity of representative ASVs was manually corroborated in the GenBank^2^ and UNITE databases. Sequences belonging to the ITS2 of the insects, mites, and plants were discarded. Sequences of all libraries were normalized in MetagenomeSeq v.1.22.0 using the method of cumulative sum scaling (Paulson et al., 2013). Libraries were homogenized at the same sample size (through multiple rarefaction) considering the library with the lowest number of sequences.

### Characterization of α- and β-diversity of mycetangial fungal assemblages

The sampling coverage was determined using rarefaction curves and the Good’s coverage estimator in QIIME2. The relative abundances of ASVs were visualized at the genus level in a heatmap developed in the CIMminer platform^3^ and associated to a dendrogram built by the UPGMA method using the Bray-Curtis index in QIIME2.

To calculate the α-diversity within these fungal assemblages, we estimated species richness using the observed ASV number and Chao1 index and species diversity with Shannon, Simpson, and Simpson’s Reciprocal indexes in QIIME2. Pairwise differences in the diversity indices between libraries were evaluated using Kruskal-Wallis test in QIIME2. Finally, to compare fungal assemblages in a multidimensional space, a principal coordinate analysis (PCoA) using Bray-Curtis dissimilarity matrix and an analysis of similarities (ANOSIM) were performed considering 999 random permutations in QIIME2.

A core mycobiome of mycetangia from the species of the *D. frontalis* complex was defined using two cut-offs in QIIME2. The first (strict core) consist in ASVs present > 70% of all libraries; the second is made up of ASVs present between 50 and 70% of libraries (relaxed core).

### Specificity of fungal assemblages to beetle species

A maximum likelihood phylogeny was inferred in PhyML 3.0 in the ATGC Montpellier Bioinformatics platform for the species of the *D*. *frontalis* complex using mtDNA cytochrome oxidase I sequences deposited in NCBI GenBank (accession no. AF60001.1 *D*. *adjunctus*, AF06999.1 *D*. *barberi*, AF068002.1 *D*. *brevicomis*, AF067986.2 *D*. *frontalis*, AF067988.1 *D*. *mexicanus*, KT364536.1 *D*. *mesoamericanus*, KT364538.1 *D. vitei*). Sequence AF067999.1 of *D*. *brevicomis* from Colorado, United States was considered as *D*. *barberi*, because this species was recently removed from its synonymy with *D*. *brevicomis* based on morphological, molecular, and chemical ecology evidence which indicates that populations from Colorado, Nevada, Utah, Arizona, New Mexico, and Texas correspond to *D*. *barberi* (Bracewell et al., 2018; Valerio-Mendoza et al., 2019; Sullivan et al., 2021). Sequences were aligned with Clustal X v.2.1 (Larkin et al., 2007) and manually edited in Seaview v.4.0.5 (Gouy et al., 2010). The best nucleotide substitution model that fit the sequence set was determined in jModelTest v.2.1.10 (Darriba et al., 2012), being GTR + I according Akaike information criterion (k = 20, −Ln = 3018.98366, AIC = 6077.96732). A bootstrap test using 1000 pseudoreplicates was carried out to evaluate the robustness of the clusters. *D*. *adjunctus* was used as the outgroup. A dendrogram of fungal assemblages was built by the unweighted arithmetic average clustering method (UPGMA) in QIIME2 using Bray-Curtis dissimilarity index. The topologies of the bark beetles and fungal assemblages were reconciled in Jane v.4.0 (Conow et al., 2010), mixing general kinds of coevolutionary events, such as cospeciation, host-switching, duplication, etc., and finding the best reconstructions by minimizing the global cost. Two models were tested, edges (edge) and nodes (node) following different cost schemes using 100 generations and a population size of 50: Cospeciation = 0, Duplication = 1, Duplication and Host switching = 2, Loss = 1, Failure to diverge = 1. The tanglegram and randomizations of the tips of the trees and the fungal assemblage topology was reconstructed after 1000 generations and considering a population size of 1000. The significance of the association was between distance matrices was evaluated using Mantel test after 1000 permutations in PAST v.3.11 (Hammer et al., 2001).

### Ecological network analysis and trophic mode

Fungal co-occurrence network from mycetangial assemblages from species of the *D*. *frontalis* complex was generated in MENA platform (Deng et al., 2012, accessed 29 March 2022)^4^ based on random matrix theory. For this analysis, we used those ASVs obtained after of the filter process and annotated to order, class or genus and whose reads numbers were > 20. Topological properties as modularity, clustering coefficient, average path length, graph density, and average degree of network were also estimated in MENA. The Spearman correlation test with a significance value of < 0.05 was used for the construction of co-occurrence network, which was visualized in Cytoscape v.3.9.1 (Shannon et al., 2003). To evaluate the empirical network and to identify mycobiota interactions that were due to non-random, a random network was generated from the empirical structure pattern of mycetangial assemblage and tested by power-law distribution. The random network was constructed based on the Maslov-Sneppen method in MENA, which kept numbers of nodes (mycetangial taxa) and edges (connections) unchanged, but rewired positions of all links in the network. Mycetangia networks from the species of the *D*. *frontalis* complex were manually extracted from the global network to visualize the ASVs co-occurrence in each bark beetle species. Lastly, the functional guild and trophic mode of the ASVs were searched using the FUNGuild database v.1.0 (Nguyen et al., 2016), and manually completed using specialized literature and USDA fungal Database (Farr and Rossman, 2015).

## Results

### Sequencing data

A total of 3,361,885 reads were obtained from the 21 libraries analyzed. From these, 426,410 were recovered after quality control and 288,143 were used for the analysis after rarefaction. The rarefaction curves analysis and values obtained with the Good’s coverage estimator (> 99%) indicated an appropriate sampling effort for all samples (Supplementary Figure 1). A total of 182 ASVs were defined, the lowest number of observed ASVs was 10 in *D*. *barberi* and the highest was 40 in *D*. *mexicanus* (Supplementary Table 1).

### Composition of fungal assemblages

**FIGURE 1:**
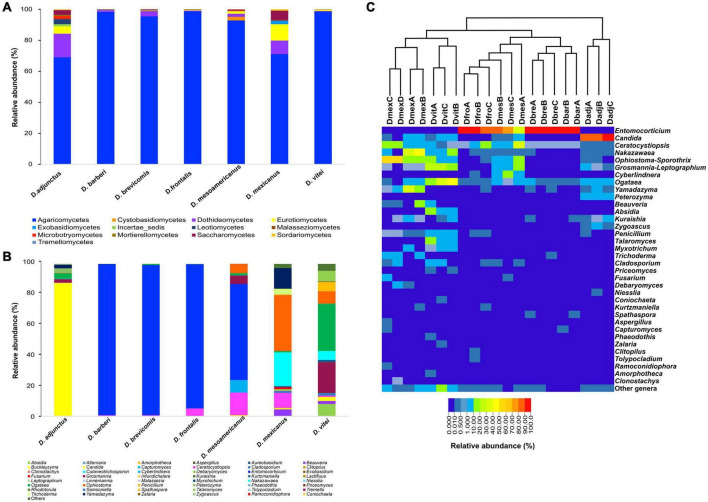
Fungal diversity and relative abundance of mycetangial fungi from the species of the *D*. *frontalis* complex. Biological replicates of each beetle species were grouped in **(A,B)**, but not in **(C)**. **(A)** Bar diagram illustrating mycetangial fungi at the class level. **(B)** Bar diagram showing mycetangial fungi at the genus level. **(C)** Heat map showing mycetangial fungi per library at the genus level by beetle species, the 21 libraries were grouped by the unweighted pair group method with arithmetic mean (UPGMA) using the Bray-Curtis dissimilarity matrix; red color represent high abundance and strong blue low abundance.

In the seven species of the *D. frontalis* complex, a total of four phyla, 13 classes, 25 orders, 39 families, and 48 genera were identified. Basidiomycota was the most abundant phylum with 50.86% of relative abundance (RA) in all samples, followed by Ascomycota (47.99%), Zygomycota (1.13%) and Mortierellomycota (< 0.01%). Basidiomycota was more abundant in *D*. *barberi* (RA = 99.28%), *D*. *brevicomis* (RA = 98.59%), *D*. *frontalis* (RA = 94.54%) and *D*. *mesoamericanus* (RA = 63.23%). Ascomycota was particularly abundant in *D*. *adjunctus* (RA = 99.98%), *D*. *mexicanus* (RA = 99.88%) and *D*. *vitei* (RA = 91.75%). Zygomycota and Mortierellomycota were only present in *D*. *vitei* (RA = 7.2%; RA = 0.04%). At the class level, Agaricomycetes was mainly abundant in all insect species, while Dothideomycetes, Eurotiomycetes, and Saccharomycetes were well represented in *D*. *adjunctus* and *D*. *mexicanus* (Figure 1A and Supplementary Figure 2A). In all libraries, the most identified orders were Corticiales, Saccharomycetales, and Ophiostomatales (Supplementary Figure 2B). Saccharomycetales and Ophiostomatales were mainly detected in *D*. *adjunctus*, *D*. *mesoamericanus*, *D*. *mexicanus*, and *D*. *vitei*, while Corticiales was present in high abundance in *D*. *barberi*, *D*. *brevicomis*, *D*. *frontalis*, and *D*. *mesoamericanus* (Supplementary Figure 2C).

At the genus level, 33 filamentous fungi and 15 yeasts were identified (Figure 1B). The most abundant genera found in the 21 libraries were *Entomocorticium*, *Candida*, *Ophiostoma-Sporothrix*, *Ogataea*, *Nakazawaea*, *Yamadazyma*, *Ceratocystiopsis*, *Grosmannia-Leptographium*, *Absidia*, and *Cyberlindnera*. Other genera had relative abundances < 1% and low prevalence in all bark beetle species (Figures 1B,C). The genus *Entomocorticium* was dominant (RA = 50.95%) in the libraries from *D*. *barberi*, *D*. *brevicomis*, *D*. *frontalis*, and *D*. *mesoamericanus*. Other taxa with relative abundances between 13 and 87% were *Candida* in *D*. *adjunctus*, *Ogataea* in *D*. *vitei*; *Ophiostoma-Sporothrix*, *Nakazawaea*, and *Yamadazyma* in *D*. *mexicanus*; *Ceratocystiopsis* in *D*. *mesoamericanus* and *Grosmannia-Leptographium* in *D*. *vitei*. Lastly, the genera *Nakazawaea*, *Ophiostoma-Sporothrix*, *Penicillium*, *Talaromyces*, and *Grosmannia-Leptographium*, showed low relative abundances between 4.5 and 20% in *D*. *vitei*, *Ophiostoma-Sporothrix* in *D*. *mesoamericanus*, and *Ceratocystiopsis* in *D*. *frontalis* and *D*. *mexicanus*.

### α and β-diversity of fungal assemblages

The fungal assemblages of *D*. *vitei* showed the highest richness (Chao1 = 29 ± 6.08) and diversity (Shannon = 3.28 ± 0.27; Simpson = 0.83 ± 0.04), while the fungal assemblages of *D*. *brevicomis* had the lowest values of richness (Chao1 = 9 ± 1.00) and *D*. *barberi* had the lowest diversity (Shannon = 0.43 ± 0.01; Simpson = 0.12 ± 0.01). The number of dominant species in the fungal assemblages varied from five in *D*. *vitei* to one in *D*. *barberi* and *D*. *frontalis* (Table 2). Kruskal-Wallis test showed significant differences in richness (Chao1) and diversity (Shannon, Simpson) in at least one pair of the fungal assemblages from the species of the *D*. *frontalis* complex, except in *D. barberi* which did not show differences with other assemblage (Table 2). Highlighting the fungal assemblage from *D*. *brevicomis* as the most different in richness, and those from *D*. *frontalis*, *D*. *mexicanus*, and *D*. *vitei* as the most diverse (Table 2).

**TABLE 2:** Amplicon sequence variant (ASVs) richness and diversity indexes of the mycetangial fungi assemblages from the species of the *Dendroctonus frontalis* complex.

Species	Observed ASVs	Chao1	Shannon diversity index	Simpson diversity index (1-D)	Simpson reciprocal index (1/D)
*D*. *adjunctus* (a)	21.33 ± 4.04 ^c^	21.33 ± 4.04 ^c^	1.58 ± 0.22 ^d,f,g^	0.45 ± 0.05 ^c,d,f,g^	2
*D*. *barberi* (b)	10.50 ± 0.71	10.50 ± 0.71	0.43 ± 0.01	0.12 ± 0.01	1
*D*. *brevicomis* (c)	9.00 ± 1.00 ^a,d,e,f,g^	9.00 ± 1.00 ^a,d,e,f,g^	1.25 ± 0.17 ^d,e,f,g^	0.53 ± 0.03 ^a,d,f,g^	2
*D*. *frontalis* (d)	16.66 ± 5.13 ^c,g^	16.83 ± 5.35 ^c,g^	0.82 ± 0.20 ^a,c,e,f,g^	0.24 ± 0.07 ^a,c,e,f,g^	1
*D*. *mesoamericanus* (e)	22.33 ± 1.53 ^c^	22.33 ± 1.53 ^c^	2.21 ± 0.94 ^c,d^	0.58 ± 0.24 ^d^	2
*D*. *mexicanus* (f)	22.00 ± 5.83 ^c^	22.00 ± 5.83 ^c^	2.52 ± 0.36 ^a,c,d,g^	0.70 ± 0.08 ^a,c,d,g^	3
*D*. *vitei* (g)	29.00 ± 6.08 ^c,d^	29.00 ± 6.08 ^c,d^	3.28 ± 0.27 ^a,c,d,f^	0.83 ± 0.04 ^a,c,d,f^	5

Data are means ± *SD* of three replicates, except to *D*. mexicanus (4) and *D*. barberi (2). Superscript letters indicate significant differences between pairs of species (*p* < 0.05) estimated with Kruskal-Wallis test.

**FIGURE 2:**
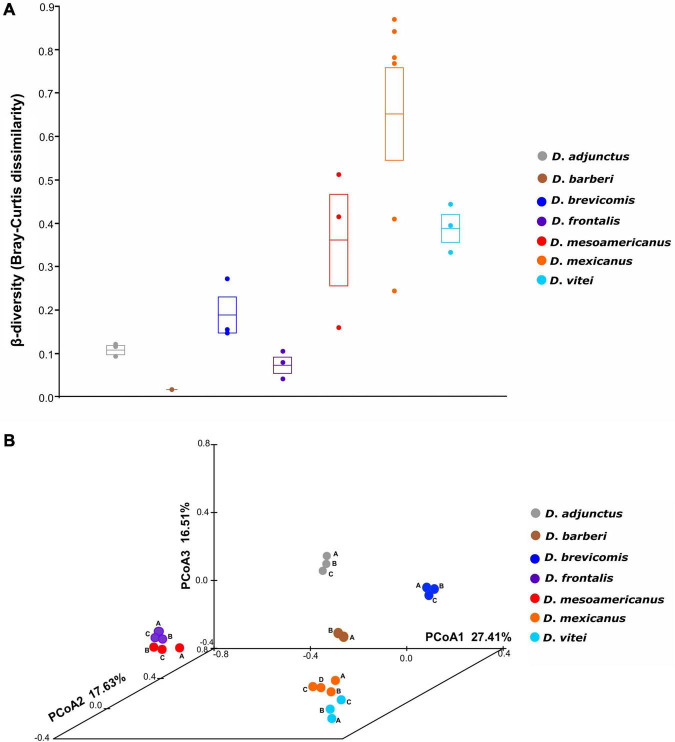
Beta-diversity and principal coordinate analysis (PCoA) based on Bray-Curtis dissimilarity. **(A)** Significant differences among in the mycangial fungal communities from the species of the *D*. *frontalis* complex (ANOSIM, *p* = 0.001). The circles represent pairwise Bray-Curtis dissimilarity values between biological replicates from species of the *D. frontalis* complex; the transversal line and ends in the box represent the mean and its standard error. **(B)** PCoA plot depicting variation in a multidimensional space of mycetangial fungal assemblages from the species of the *D*. *frontalis* complex; the first three PCoA coordinates explained 61.55% of the total observed variation; dots of the same color represent biological replicates (A, B, C) of the same bark beetle species, except for *D*. *mexicanus* (A, B, C, D) and *D*. *barberi* (A, B).

Significant differences (ANOSIM, *p* = 0.001) were found between the mycetangial assemblages of the different species of the *D*. *frontalis* complex using Bray-Curtis dissimilarity matrix (Figure 2A). The first three coordinates of the PCoA explained 61.55% of the total observed variation (Figure 2B). A clear spatial segregation of libraries corresponding to *D*. *adjunctus*, *D*. *barberi*, and *D*. *brevicomis* species was observed in the multidimensional space, but not between close species *D*. *frontalis* and *D*. *mesoamericanus*, as well as *D*. *mexicanus* and *D*. *vitei.*

The absence of a strict core was evident because only the genus *Ceratocystiopsis* was shared among 21 libraries of seven species (represented by different ASVs). Only two genera, *Nakazawaea* and *Ogataea*, were found in the relaxed core, because they were recovered in at least 70% of libraries, but also represented with different ASVs.

### Fungal assemblages and their association with the species of the *Dendroctonus frontalis* complex

**FIGURE 3:**
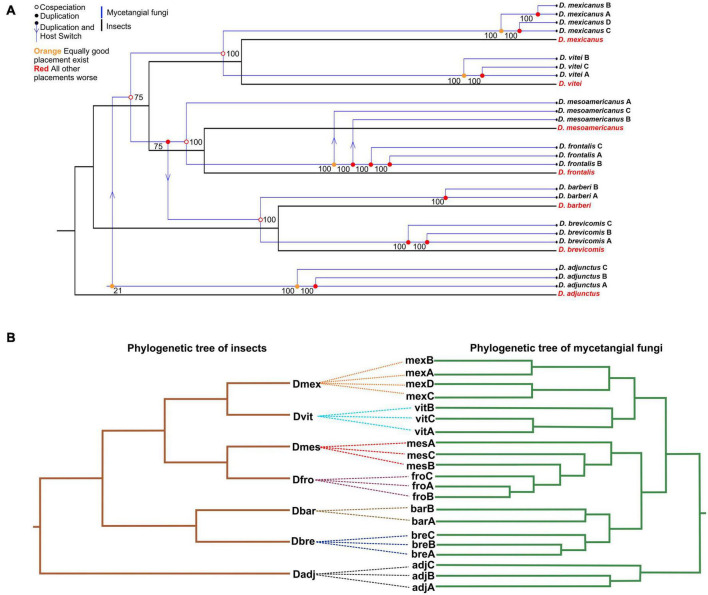
The tanglegram of fungal assemblages and species of the *D*. *frontalis* complex. **(A)** Topological reconciliation of bark beetles and fungal assemblages. Four cospeciation events were found, the first between *D*. *mexicanus* and *D*. *vitei*; the second between *D*. *mesoamericanus* and *D. frontalis*; the third between groups *D*. *mexicanus* + *D*. *vitei* and *D*. *mesoamericanus* + *D*. *frontalis*; and the last between *D*. *barberi* and *D*. *brevicomis*. Four duplication and host switch events were detected between *D*. *frontalis* and *D*. *mesoamericanus* (2), between groups *D*. *mesoamericanus* + *D*. *frontali*s and *D*. *barberi* + *D*. *brevicomis* (1), and the last between *D*. *adjunctus* and the group of *D*. *mexicanus* + *D*. *vitei* + *D*. *mesoamericanus* + *D*. *frontalis* (1). The reconciliation of topologies (*p* = 0.01) and association between distance matrices (Mantel test *r* = 0.7093, *p* = 0.003) were significant. **(B)** ML-phylogeny from the species of the *D*. *frontalis* complex inferred using mtDNA COI sequence (AF60001.1 *D*. *adjunctus*, AF06999.1 *D*. *barberi*, AF068002.1 *D*. *brevicomis*, AF067986.2 *D*. *frontalis*, AF067988.1 *D*. *mexicanus*, KT364536.1 *D*. *mesoamericanus*, KT364538.1 *D*. *vitei*). The model best was GTR + I *sensu* Akaike criterion (k = 20, −Ln = 3018.98366, AIC = 6077.96732). Bootstrap values are shown upper nodes. *D*. *adjunctus* was used as outgroup. Dendrogram of fungal assemblages built using UPGMA and Bray-Curtis dissimilarity index.

The tanglegram showed a significant global congruence between fungal assemblages and species of the *D*. *frontalis* complex (Figures 3A,B). Four cospeciation events and four duplication and host switch events were inferred. The first cospeciation event was between *D*. *mexicanus* and *D*. *vitei*; the second between *D*. *mesoamericanus* and *D. frontalis*; the third between groups *D*. *mexicanus* + *D*. *vitei* and *D*. *mesoamericanus* + *D*. *frontalis*; and the last between *D*. *barberi* and *D*. *brevicomis*. The four events of duplication and host switch were distributed between *D*. *frontalis* and *D*. *mesoamericanus* (2), between groups *D*. *mesoamericanus* + *D*. *frontali*s and *D*. *barberi* + *D*. *brevicomis* (1), and the last between *D*. *adjunctus* and the group of *D*. *mexicanus* + *D*. *vitei* + *D*. *mesoamericanus* + *D*. *frontalis* (1). Topological reconciliation showed that the species of the *D*. *frontalis* complex has potentially exclusive mycetangial assemblages, because the ASVs are not completely shared. The reconciliation of topologies (*p* = 0.01) and association between distances matrices from bark beetles and mycetangial assemblages was statistically significant (Mantel test *r* = 0.7093, *p* = 0.003).

### Ecological network analysis of mycetangial assemblages

**FIGURE 4:**
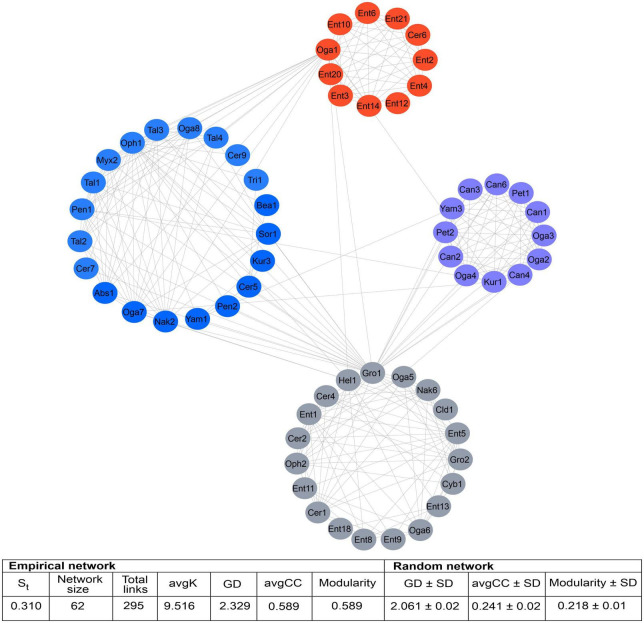
Global network of the mycetangial fungi assemblages from the species of the *D*. *frontalis* complex. It was built with 105 ASVs whose reads > 20 reads, which include to one class, one order,13 genera of filamentous fungi and 9 yeasts. ASVs showed a network contained 62 nodes connected by 295 edges, of which 50.85% were positive connections (mutualistic or commensalistic interactions) and 49.15% negative connections (antagonistic interactions). Four modules integrated the general network. The module 0 included the ASVs from *D*. *mexicanus* and *D*. *vitei*, the module 1 the ASVs from *D*. *adjunctus*, the module 2 the ASVs from *D*. *barberi* and *D*. *brevicomis*, and the module 3 the ASVs from *D*. *frontalis* and *D*. *mesoamericanus*. The connectivity distributions in this general network followed the power-law model (*R*^2^ = 0.013). The values of modularity, average connectivity, average path distance, and average clustering coefficient were significantly higher that of a random network (*p* < 0.05) and they are shown in the framework under the figure. Filamentous Fungi: Abs, *Absidia*; Bea, *Beauveria*; Cer, *Ceratocystiopsis;* Cld, *Cladosporium;* Ent, *Entomocorticium*; Gro, *Grosmannia-Leptographium*; Hel, Helotiales; Myx, *Myxotrichum;* Oph, *Ophiostoma-Sporothrix;* Pen, *Penicillium;* Sor, Sordariomycetes; Tal, *Talaromyces;* Tri, *Trichoderma.* Yeast: Can, *Candida;* Cyb, *Cyberlindnera;* Kur, *Kuraishia;* Nak, *Nakazawaea;* Oga, *Ogataea;* Pet, *Peterozyma*; Yam, *Yamadazyma*.

**FIGURE 5:**
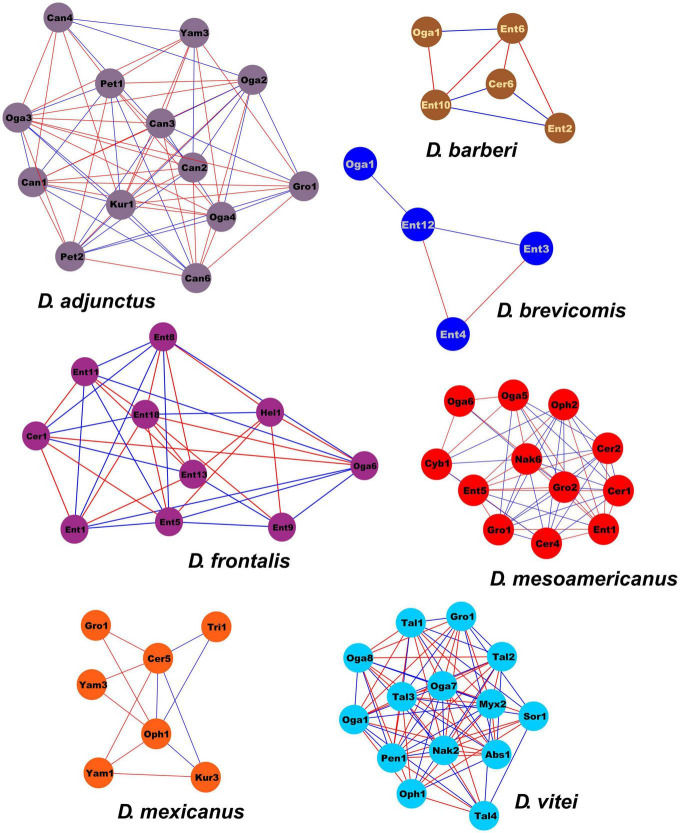
Individual network extracted from global network of the mycetangial fungi from each species of the *D*. *frontalis* complex species. The nodes are colored in each module according to the bark beetle species from which ASVs were identified. Individual networks were integrated by different ASV numbers. Blue lines indicate positive interactions (mutualism or commensalism), while a red lines indicates negative interactions (antagonism). Filamentous Fungi: Abs, *Absidia*; Bea, *Beauveria;* Cer, *Ceratocystiopsis;* Cld, *Cladosporium;* Ent, *Entomocorticium*; Gro, *Grosmannia-Leptographium*; Hel, Helotiales; Myx, *Myxotrichum*; Oph, *Ophiostoma-Sporothrix*; Pen, *Penicillium*; Sor, Sordariomycetes; Tal, *Talaromyces*; Tri, *Trichoderma.* Yeast: Can, *Candida*; Cyb, *Cyberlindnera*; Kur, *Kuraishia*; Nak, *Nakazawaea;* Oga, *Ogataea;* Pet, *Peterozyma*; Yam, *Yamadazyma*.

From 182 ASVs recovered on a total of 288,143 sequences in the DNA metabarcoding analysis, only 105 had > 20 reads. The association of these 105 ASVs showed a network containing 62 nodes connected by 295 links, of which 50.85% were positive connections (e.g., mutualistic or commensalistic interactions) and 49.15% were negative connections (e.g., antagonistic interactions). Nodes were represented by one class, one order, 11 genera of filamentous fungi and 7 yeasts. Four modules were integrated in the global network. The module 0 included ASVs from *D*. *mexicanus* and *D*. *vitei*, represented by four and six filamentous fungi genera, respectively, and two yeasts genera each one. The module 1 was integrated by ASVs from *D*. *adjunctus* represented by one filamentous fungi genus and five yeasts genera. The module 2 was formed by ASVs from *D*. *barberi* and *D*. *brevicomis*, represented by two and one filamentous fungi genera, respectively, and one yeast each one. Lastly, the module 3 was formed by ASVs from *D*. *frontalis* and *D*. *mesoamericanus*, defined by two and four filamentous fungi genera and one and three yeasts, respectively. The ASVs belonging to *Ogataea* (Oga1) and *Grosmannia* (Gro1) had > 10 interactions with ASVs of others modules (Figure 4). The connectivity distribution in this network followed the power-law model (*R*^2^ = 0.013). The values of modularity (*M* = 0.589), average connectivity (avgK = 9.516), average path distance (GD = 2.329), and average clustering coefficient (avgCC = 0.778) were significantly higher than the random network values (*M* = 0.218, GD = 2.061, avgCC = 0.241), suggesting that the mycetangial ASVs assemblage was non-random (*p* < 0.05) (Figure 4). Individual networks were integrated by different ASV numbers. For example, *D*. *brevicomis*, *D*. *barberi*, and *D*. *mexicanus* had < 9 ASVs, whereas *D*. *adjunctus*, *D*. *frontalis*, *D*. *mesoamericanus*, and *D*. *vitei* had ≥ 10. These networks were almost entirely integrated by different ASVs, with a few exceptions, including the ASVs of Cer1, Ent1,5, Oga1,6, Oph1, Gro1, and Yam3, which were shared among the bark beetle species (Figure 5 and Supplementary Table 2). Based on the number of positive and negative interactions (edges), the dominant condition of some ASVs in the individual networks was evident, highlighting those from the genus *Candida* (Can) in the *D*. *adjunctus* network; *Entomocorticium* (Ent) in the networks of *D*. *barberi*, *D*. *brevicomis*, and *D*. *frontalis*; *Ceratocystiopsis* (Cer), *Yamadazyma* (Yam), and *Talaromyces* (Tal) in the networks of *D*. *mesoamericanus*, *D*. *mexicanus*, and *D*. *vitei*, respectively (Figure 5 and Supplementary Table 2). All these results suggest that the fungal assemblage networks associated to the species of the *D*. *frontalis* complex was non-randomly distributed and had a highly connected topological structure.

The total ASVs (182) were assigned into seven trophic modes at the following frequencies: symbiotroph/saprotroph (30.77%), followed by saprotroph (27.47%), pathotroph (19.79%), pathotroph/saprotroph/symbiotroph (11.54%), pathotroph/saprotroph (3.85%), pathotroph/symbiotroph (2.75%), symbiotroph (1.65%), and unassociated (2.20%). The guild of these fungi and yeast genera fell into the categories plant or animal pathogen, animal symbionts, wood saprotrophs, and less often lichen and fungal parasite (Supplementary Table 3).

## Discussion

This is the first comprehensive study on the mycobiome of pronotal mycetangia from the species of the *Dendroctonus frontalis* complex. Based on Good’s coverage (>99%) and rarefaction curves, sampling effort was adequate. Our findings show the presence of fungal assemblages in the mycetangia of these beetles integrated both by filamentous fungi and yeasts, which varies among bark beetle species. This diversity is apparently limited to 33 genera of filamentous fungi and 15 genera of yeasts belonging to phyla Basidiomycota (50.86%), Ascomycota (47.99%), Zygomycota (1.13%), and Mortierellomycota (<0.01%) using ITS2 region. These results should be interpreted cautiously, because the intraspecific variation among ribosomal unit of the cluster rRNA loci, which has multiple copies in the fungi genome of the same species, could inflate the estimates of ASV richness and its abundance (Taylor et al., 2016; Lavrinienko et al., 2021). Thus, the inclusion of additional molecular markers would facilitate a better characterization of mycetangial assemblages.

The results indicate that the phylum Basidiomycota was more abundant than Ascomycota, in contrast to results reported for other bark beetles (Harrington, 2005), ambrosial insects (Henriques et al., 2006), and other Coleoptera (Biedermann and Vega, 2020) where Ascomycota is the dominant phylum. The presence of Mucoromycota (= Zygomycota) and Mortierellomycota are reported for the first time to be associated with the mycetangium from the species of the *D*. *frontalis* complex. Zygomycota has also been reported from the gut and galleries of *D. armandi* and *D*. *valens* in China, where they are invasive (Hu et al., 2015) and Mortierellomycota from galleries and epimycobiota of other Coleoptera (Wang et al., 2020; Held et al., 2021). Likewise, the basidiomycetes *Entomocorticium*, and ascomycetes *Candida*, *Ophiostoma-Sporothrix*, *Ogataea*, *Nakazawaea*, *Yamadazyma*, *Ceratocystiopsis*, *Grosmannia-Leptographium*, and *Cyberlindnera* were abundant in mycetangia.

Analysis of α-diversity revealed that the composition of fungal assemblages, as well as the relative abundance of their members, vary widely among species of the *D*. *frontalis* complex. Mycetangial assemblages of *D*. *adjunctus, D*. *mexicanus, D*. *mesoamericanus*, and *D*. *vitei* were more diverse than *D*. *barberi*, *D*. *brevicomis*, and *D*. *frontalis*. Significant changes in the assemblage structure are evident, as exemplified by *Entomocorticium* that is abundant and apparently exclusive to *D*. *barberi*, *D*. *brevicomis*, *D*. *frontalis*, and *D*. *mesoamericanus*, whereas *Ogataea* and *Grosmannia-Leptographium* are more prevalent in *D*. *vitei*, but *Candida* was the most abundant in *D. adjunctus.* Other genera such as *Ceratocystiopsis* and *Ophiostoma-Sporothrix* are also abundant, but not exclusive of any beetle species indicating that some fungal taxa are highly specific to their host beetles whereas others are likely generalists. Alternatively, some fungal associations may predate the evolutionary divergence of beetle taxa and may have been retained as symbionts across that divergence.

Differences in α-diversity of the fungal assemblages across beetle species may be due to several factors. For example, the development and ultrastructural organization of the mycetangium varies among species in the *D*. *frontalis* complex (Barras, 1967; Happ et al., 1971; Yuceer et al., 2011). This could indicate variable microenvironmental conditions associated with the mycetangia of different beetle species, including selective factors such as gas exchange, temperature, and redox potential (Six and Bentz, 2007; Bleiker and Six, 2009; Addison et al., 2013; Dysthe et al., 2015; Six and Klepzig, 2021). In addition, mycetangial glandular cells provide molecules to regulate the growth of specific fungi and bacteria (Francke-Grossman, 1967; Barras and Perry, 1971; Happ et al., 1971; Yuceer et al., 2011) or produce chemical secretions that inhibit the growth of unspecific fungi or favor the presence of symbiotic fungi (Schneider and Rudinsky, 1969; Happ et al., 1971; Paine and Birch, 1983).

Differences found in β-diversity are due to the absence of both dominant and marginal members in assemblages (Figure 1B). This explains the absence of a strict core mycobiome for species of the *D*. *frontalis* complex as a whole. On another hand, the presence of a relaxed minimal core consisting of *Ceratocystiopsis*, *Nakazawaea*, and *Ogataea*, along with the presumed exclusivity of the assemblages observed in the tanglegram, suggest that a core for individual species may exist. This hypothesis could be tested in future studies incorporating samples of these bark beetles from other localities, as the distribution of both insects and symbionts is not random but depending on ecological opportunities and selective pressures in geographic space, which eventually could modify the interaction among them, as well as the integration of minimal assemblages or the membership of some symbionts within the core mycobiome.

Members of this core may be playing a relevant ecological role and complex. For example, *Ceratocystiopsis* is a pathogenic fungus to pines and has been considered important to overcome the defensive system of healthy trees during beetle attacks, potentially facilitating successful colonization (Harrington, 1993). However, phytopathogenicity is likely not the evolutionary driver behind these associations and pathogenic fungi may be rarer associates than ecologically neutral symbionts (Six and Wingfield, 2011). Complex ecological roles might not be exclusive of the filamentous fungi; for instance, because it has demonstrated that *Ogataea* yeast can favor the growth of filamentous fungi, but also inhibit other fungi (Davis et al., 2011). Thus, collectively, the functions developed by the core members may have crucial importance in the life cycle of bark beetle species.

Despite their abilities to perform diverse functions to the benefit of these insects, the overrepresentation of certain fungi (e.g., *Entomocorticium*, *Ophiostoma-Sporothrix*, *Ceratocystiopsis*, and *Grosmannia-Leptographium*) in the species of the *D. frontalis* complex suggests that they are not randomly acquired even though they are transmitted horizontally, and their spores are passively incorporated into mycetangia (Beaver, 1989). This is also applicable to yeasts (e.g., *Ogataea*, *Candida, Nakazawaea*, *Yamadazyma*, *Cyberlindnera*) whose acquisition is horizontal, as suggested by their ubiquity in all developmental stages and body structures including mycetangia of these and other bark beetles (Rivera et al., 2009; Davis, 2015). The FUNGuild analysis showed that almost all filamentous fungi recorded in this study (e.g., *Ophiostoma-Sporothrix*, *Grosmannia-Leptographium*, *Beauveria*) are pathotrophs, whereas yeasts and some filamentous fungi (e.g., *Ogataea*, *Kuraishia*, *Nakazawaea*, *Yamadazyma*, *Cyberlindnera*, *Peterozyma*, *Zygoascus, Entomocorticium, Ceratocystiopsis, Penicillium*) are symbiotrophs or saprotrophs. Independently of their trophic mode and horizontal transmission, the nested structure of the global ecological network (i.e., it was more nested than the random network), in which few interacting species are compartmentalized into modules, suggesting that minimal assemblages with relatively high species redundancy are maintained, regulated, and structured within the mycetangia of these insects (Figure 4 and Supplementary Figure 3).

Although several studies have tested interaction between microbes isolated from the mycetangium (Scott et al., 2008; Oh et al., 2009; Davis et al., 2011), to our knowledge it is unknown how these assemblages are regulated and stabilized over space and time. The action of antibiotics, antifungals, mycotoxins and volatile organic compounds produced by fungi, yeasts, and bacteria, as well as by chemical secretions produced by mycetangial glands, or the immune system of ambrosia and bark beetles could be factors involved in this self-regulation (Nakashima et al., 1982; Scott et al., 2008; Oh et al., 2009; Shi and Sun, 2010). Other factors that could influence the regulation of mycetangial fungal assemblages include beneficial (mutualism, commensalism), competitive or neutral interactions of their members within integuments, as well as their potential functional roles, which could vary geographically with shifting environmental and biotic pressures.

Thus, the observed sporadic occurrence of some fungi or yeasts, as well as the possible loss of overrepresented members suggest that they are incidental or transient members of mycetangial assemblages, which could result in replacement by another member with a similar ecological niche (Six, 2012, 2019; Hofstetter et al., 2015; Bracewell et al., 2018). This functional redundancy could help to stabilize ecological role of mycetangial assemblages and guarantee the regulation of their members over space and time.

Lastly, the positive correlation between the similarity of the fungi assemblage composition and the phylogenetic relatedness of species of the *D*. *frontalis* complex observed in the tanglegram, suggest an ecological pattern of phylosymbiosis (Figure 3). This pattern, as suggested by events associated to tanglegram (cospeciation and duplication and host switch), could have been generated through the diversification from the species of the *D*. *frontalis* complex, as it was recently demonstrated in the case of mycetangial fungi associated to *D*. *brevicomis* and *D*. *barberi* (Bracewell et al., 2018). However, at shared habitats such as mycetangia, it cannot be discarded that this pattern could be raised and structured by habitat selection, where the abiotic and biotic environment into mycetangia could determinate the composition and specificity of the assemblages. Further research is required to test whether the phylosymbiosis corresponds with diversification event of these bark beetles or well if it is result non-adaptive host filtering (Groussin et al., 2020).

In summary, these findings improve our knowledge of the diversity of mycetangial assemblages in species of the *D. frontalis* complex. The isolation and culture of some fungal species has helped to clarify the nature of the relationship of individual members with *Dendroctonus* species, as well as the implications they have on the biology, ecology, and evolution of these insects. Our results add to this literature and suggest that specific minimal assemblages are maintained, regulated, and structured within the mycetangium of these insects. Analyzing these assemblages using an integrated approach, such as we do here, helps to further clarify the nutritional, physiological, and ecological roles of their members, as well as their potential interactions. This approach is a useful first step in better understanding these systems. Further work in this area is needed to analyze and better understand integral functional aspects, such as metabolite level and composition, metatranscriptomic and/or metaproteomic response of these communities within the mycetangium of their hosts, and multispecific interactions *in vitro* and *in vivo.*

## Data availability statement

The datasets presented in this study can be found in National Center for Biotechnology Information (NCBI) Bioproject database under accession number: PRJNA813135.

## Author contributions

KV-O, FR-O, and GZ conceived the work. KV-O performed the experiment. KV-O, RP-M, RG-E, TD, KS, FR-O, and GZ interpreted the result, performed the draft, and final edition of the manuscript. All authors contributed and approved the final manuscript.

## Footnotes

1
https://unite.ut.ee

2
https://blast.ncbi.nlm.nih.gov/Blast.cgi

3
https://discover.nci.nih.gov/cimminer/home.do

4
http://ieg4.rccc.ou.edu/MENA/main.cgi
