# Supplementary material for: Metabarcoding of mycetangia from the Dendroctonus frontalis species complex (Curculionidae: Scolytinae) reveals diverse and functionally redundant fungal assemblages
Source: Front Microbiol. 2022 Sep 16;13:969230. doi: 10.3389/fmicb.2022.969230 (PMC9524821; doi:10.3389/fmicb.2022.969230)
Supplement: Supplementary file 2 [file Data_Sheet_1.docx]

***Supplementary Material***

**Metabarcoding of mycetangia from the species of *Dendroctonus frontalis* complex (Curculionidae: Scolytinae) reveals diverse and functionally redundancy fungal assemblages**

**Karina Vazquez-Ortiz, Rosa María Pineda-Mendoza, Román González-Escobedo, Thomas S. Davis, Kevin F. Salazar, Flor N. Rivera-Orduña and Gerardo Zúñiga**

**Supplementary Tables**

**Supplementary Table 1.** Illumina sequencing data obtained from mycetangial assemblages from the species of the *D*. *frontalis* complex.

| **Replicates** | **Raw reads** | **Reads after quality control with DADA2** | **Observed ASVs** | **Good’s coverage (%)** |
| --- | --- | --- | --- | --- |
| *D*. *adjuntus* A | 207,203 | 15,811 | 21 | 100 |
| *D*. *adjuntus* B | 245,154 | 36,492 | 25 | 100 |
| *D*. *adjuntus* C | 214,161 | 27,228 | 17 | 99 |
| *D*. *barberi* A | 113,151 | 44,225 | 10 | 100 |
| *D*. *barberi* B | 98,274 | 45,349 | 11 | 99 |
| *D*. *brevicomis* A | 97,033 | 10,596 | 10 | 100 |
| *D*. *brevicomis* B | 182,433 | 29,935 | 8 | 100 |
| *D*. *brevicomis* C | 192,300 | 23,475 | 9 | 100 |
| *D*. *frontalis* A | 166,121 | 30,971 | 11 | 100 |
| *D*. *frontalis* B | 179,293 | 48,864 | 18 | 99 |
| *D*. *frontalis* C | 191,384 | 35,924 | 21 | 99 |
| *D*. *mesoamericanus* A | 115,682 | 6,422 | 24 | 100 |
| *D*. *mesoamericanus* B | 117,699 | 19,927 | 22 | 100 |
| *D*. *mesoamericanus* C | 125,681 | 23,238 | 18 | 100 |
| *D*. *mexicanus* A | 164,938 | 9,606 | 28 | 100 |
| *D*. *mexicanus* B | 141,396 | 4,590 | 23 | 100 |
| *D*. *mexicanus* C | 189,628 | 2,389 | 23 | 100 |
| *D*. *mexicanus* D | 109,452 | 708 | 14 | 100 |
| *D*. *vitei* A | 151,852 | 4,710 | 33 | 100 |
| *D*. *vitei* B | 176,776 | 3,320 | 22 | 100 |
| *D*. *vitei* C | 182,274 | 2,630 | 32 | 100 |

**Supplementary Table 2.** Nodes and interactions inferred in the individual ecological networks of the mycetangial assemblage from the species of the *D*. *frontalis* complex.

| **Beetle species** | **Fungal taxonomy** | **Node name** | **Positive interactions** | **Negative interactions** |
| --- | --- | --- | --- | --- |
| ***D*. *adjunctus*** | *Candida* | Can1 | 4 | 7 |
|  |  | Can2 | 4 | 5 |
|  |  | Can3 | 4 | 5 |
|  |  | Can4 | 3 | 4 |
|  |  | Can6 | 4 | 6 |
|  | *Kuraishia* | Kur1 | 4 | 8 |
|  | *Grosmannia* | Gro1 | 4 | 5 |
|  | *Ogataea* | Oga2 | 6 | 5 |
|  |  | Oga3 | 4 | 8 |
|  |  | Oga4 | 4 | 6 |
|  | *Peterozyma* | Pet1 | 4 | 6 |
|  |  | Pet2 | 6 | 4 |
|  | *Yamadazyma* | Yam3 | 2 | 6 |
| ***D*. *barberi*** | *Ceratocystiopsis* | Cer6 | 2 | 1 |
|  | *Entomocorticium* | Ent2 | 2 | 1 |
|  |  | Ent6 | 1 | 3 |
|  |  | Ent10 | 2 | 2 |
|  | *Ogataea* | Oga1 | 1 | 1 |
| ***D*. *brevicomis*** | *Entomocorticium* | Ent3 | 1 | 1 |
|  |  | Ent4 | 0 | 2 |
|  |  | Ent12 | 2 | 1 |
|  | *Ogataea* | Oga1 | 1 | 0 |
| ***D*. *frontalis*** | *Ceratocystiopsis* | Cer1 | 3 | 4 |
|  | *Entomocorticium* | Ent1 | 4 | 3 |
|  |  | Ent5 | 5 | 3 |
|  |  | Ent8 | 5 | 3 |
|  |  | Ent9 | 3 | 2 |
|  |  | Ent11 | 4 | 3 |
|  |  | Ent13 | 2 | 5 |
|  |  | Ent18 | 2 | 7 |
|  | *Ogataea* | Oga6 | 5 | 3 |
|  | Order Helotiales | Hel1 | 1 | 5 |
| ***D. mesoamericanus*** | *Ceratocystiopsis* | Cer1 | 7 | 2 |
|  |  | Cer2 | 7 | 2 |
|  |  | Cer4 | 7 | 2 |
|  | *Cyberlindnera* | Cyb1 | 4 | 2 |
|  | *Entomocorticium* | Ent1 | 1 | 8 |
|  |  | Ent5 | 1 | 8 |
|  | *Grosmannia* | Gro1 | 7 | 2 |
|  |  | Gro2 | 7 | 3 |
|  | *Nakazawaea* | Nak6 | 8 | 3 |
|  | *Ogataea* | Oga5 | 4 | 4 |
|  |  | Oga6 | 2 | 2 |
|  | *Ophiostoma* | Oph2 | 7 | 2 |
| ***D*. *mexicanus*** | *Ceratocystiopsis* | Cer5 | 3 | 3 |
|  | *Grosmannia* | Gro1 | 0 | 2 |
|  | *Kuraishia* | Kur3 | 2 | 1 |
|  | *Trichoderma* | Tri1 | 2 | 0 |
|  | *Yamadazyma* | Yam1 | 0 | 3 |
|  |  | Yam3 | 0 | 2 |
|  | *Ophiostoma* | Oph1 | 3 | 3 |
| ***D*. *vitei*** | *Absidia* | Abs1 | 5 | 6 |
|  | *Grosmannia* | Gro1 | 6 | 6 |
|  | *Myxotrichum* | Myx2 | 6 | 6 |
|  | *Nakazawaea* | Nak2 | 5 | 7 |
|  | *Ogataea* | Oga1 | 6 | 6 |
|  |  | Oga7 | 6 | 7 |
|  |  | Oga8 | 5 | 6 |
|  | *Ophiostoma* | Oph1 | 3 | 6 |
|  | Class Sordariomycetes | Sor1 | 4 | 4 |
|  | *Penicillium* | Pen1 | 5 | 7 |
|  | *Talaromyces* | Tal1 | 5 | 6 |
|  |  | Tal2 | 5 | 6 |
|  |  | Tal3 | 6 | 7 |
|  |  | Tal4 | 4 | 4 |

# Supplementary Figures

**
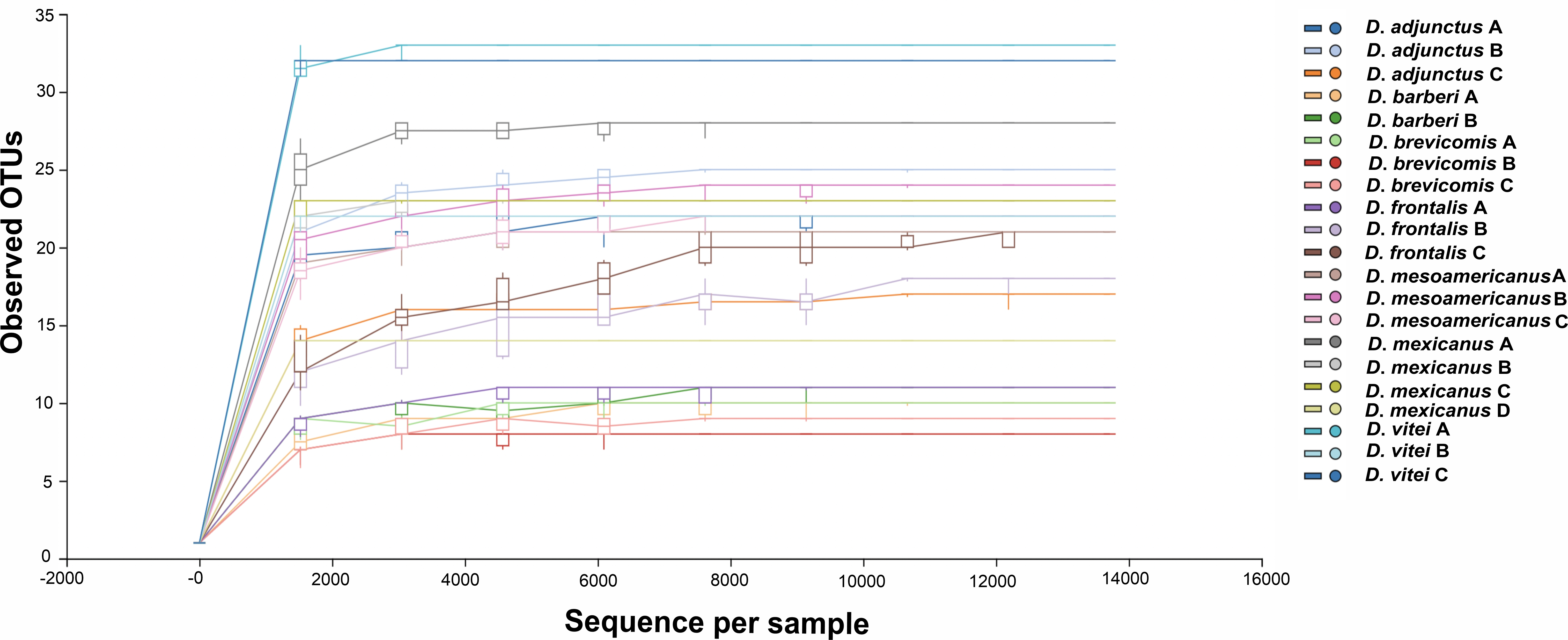
**

**Supplementary Figure 1.** Rarefaction curves of the libraries of mycetangial fungi from the species of the *D*. *frontalis* complex.

**
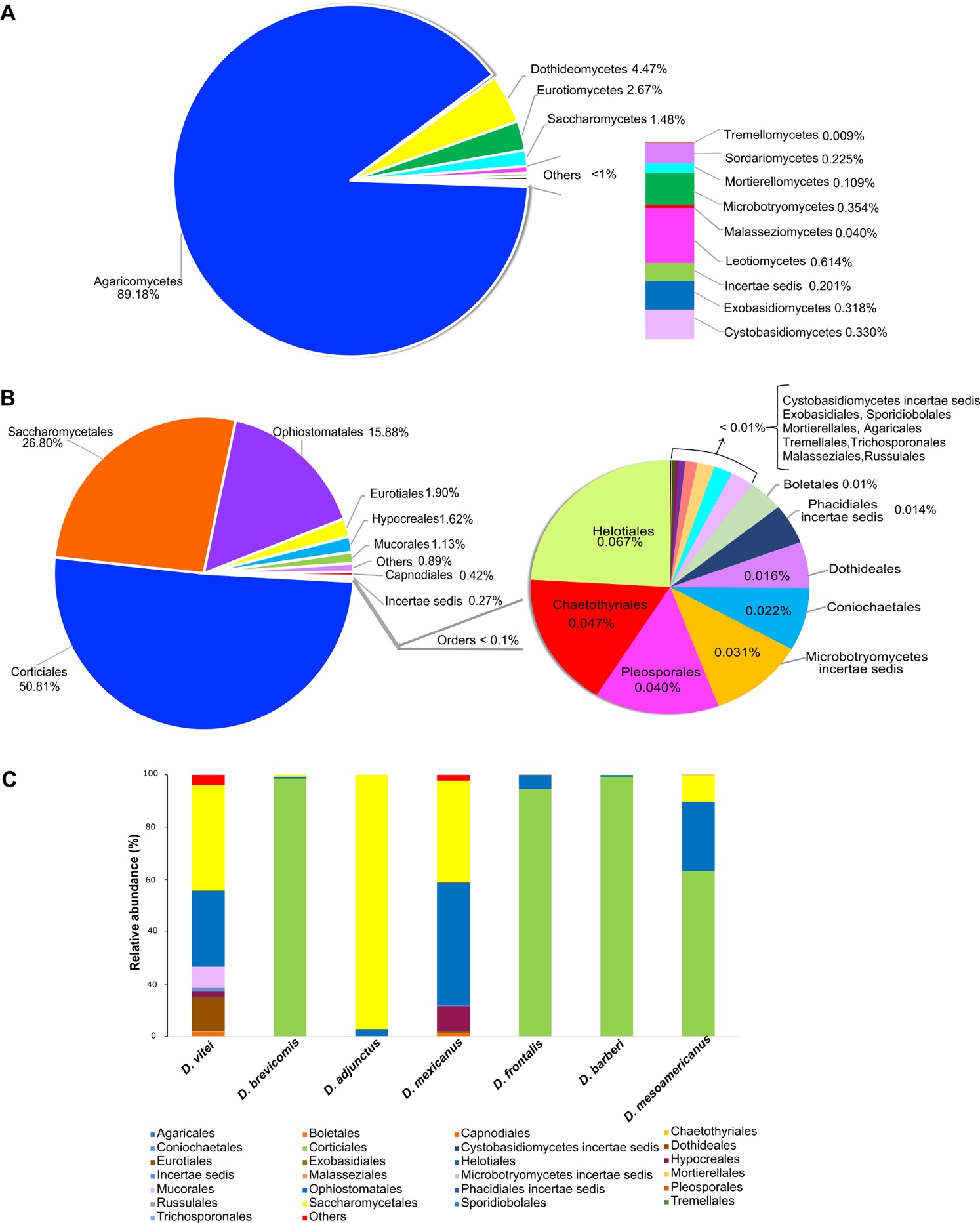
**

**Supplementary Figure 2.** Relative abundances (RA) of mycetangial assemblage from the species of the *D. frontalis* complex at the class **(A)** and order **(B** and **C** by species**)** levels.

**
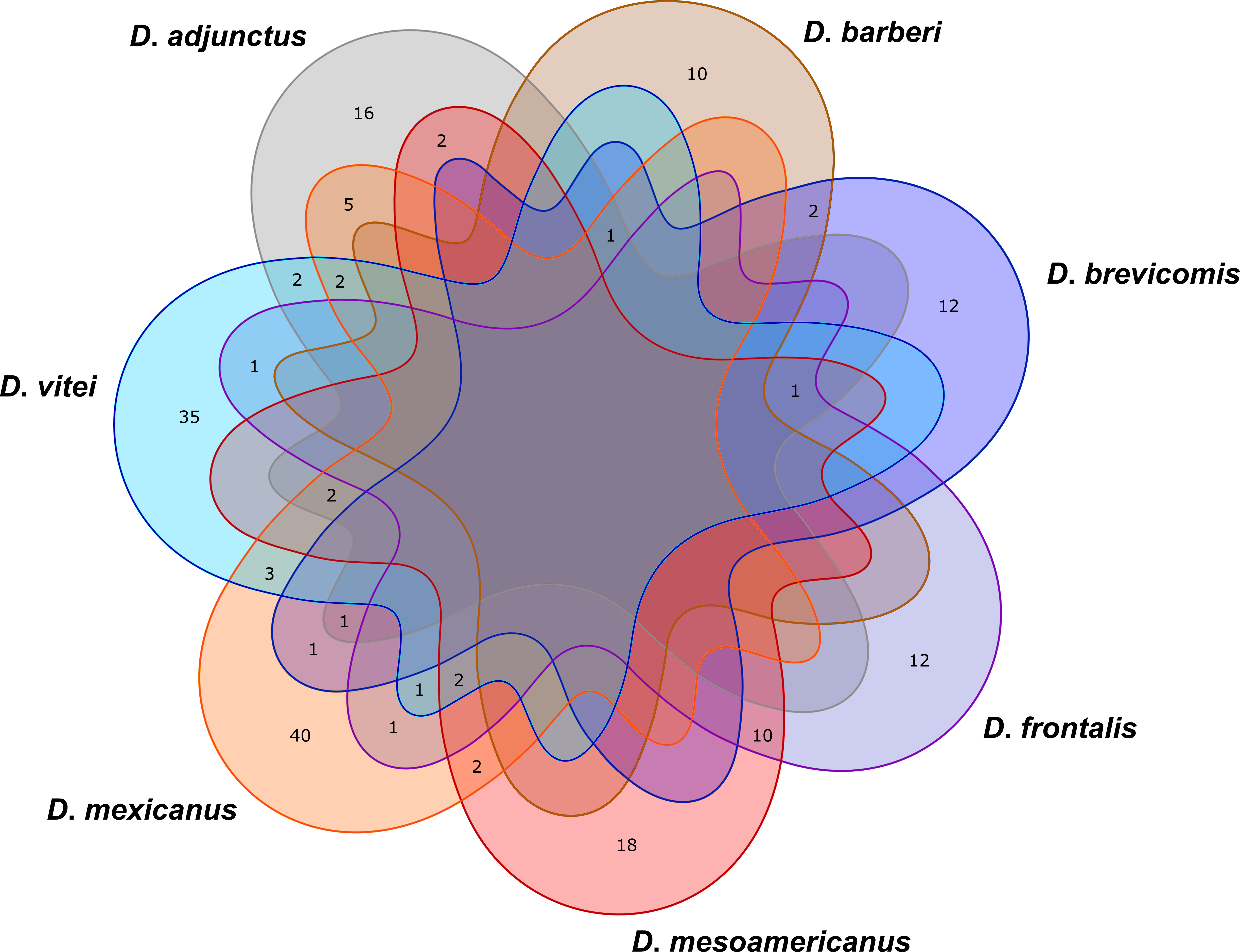
**

**Supplementary Figure 3.** Venn diagram of amplicon sequence variants (ASVs) observed in mycetangial assemblages from the species of the *D*. *frontalis* complex.
